# First and Second Metatarsophalangeal Joint Open Dislocations: A Case Report

**DOI:** 10.5704/MOJ.2211.023

**Published:** 2022-11

**Authors:** SN Pai, MM Kumar

**Affiliations:** Department of Orthopaedic Surgery, Sri Ramachandra Institute of Higher Education and Research, Chennai, India

Malays Orthop J. 2017 Mar;11(1):71-73. doi: http://dx.doi.org/10.5704/MOJ.1703.010

Dear Sir,

We read the recently published article in Malaysian Orthopaedics Journal^1^ with keen interest. The authors deserve compliments for sharing their case of dislocation of first and second metatarsophalangeal joints (MTP). However, we wanted to differ with the author on some points and add some more knowledge to the present topic of discussion on dislocation of metatarsophalangeal joints.

First, we differ on the opinion that the intact collateral ligaments make closed reduction of metatarsophalangeal joint impossible. The most common reason for failed closed reduction of MTP joint is interposed soft tissue. The volar plate is responsible for this in most instances^2^. Hence, interposed tissue rather than intact collateral ligaments is the reason for failed closed reduction. Chefik et al state that the ability to reduce the dislocation by non-operative measures depends largely on the type of dislocation and involvement of the sesamoid complex instead^3^.

Second, the authors incised the lateral collateral ligament prior to removal to all interposed tissue, which we believe might not have been warranted. Most reported cases of MTP dislocation with failed closed reduction, even with intact collateral ligaments, have been open reduced with only removal of interposed tissue^4,5^. Younis et al describe the technique of open reduction with intact collateral ligaments by applying axial traction and inserting a Blunt Hohmann retractor from under the inter-sesamoid ligament on the head of the first metatarsal to lever the proximal phalanx back in its place on the head of first metatarsal^6^.

Third, as was encountered by the authors, it is not uncommon for the second MTP joint to remain dislocated after the relocation of the first MTP joint^4^.

**Fig. 1.**
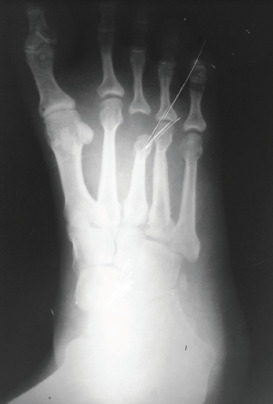
Radiograph of foot showing dislocation of 3rd MTP joint.

**Fig. 2.**
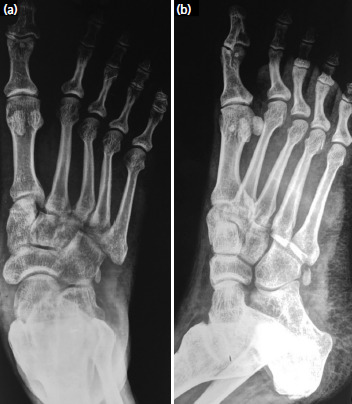
(a) Anteroposterior and (b) oblique radiographs of foot six weeks after closed reduction and immobilisation showing normally aligned 3rd MTP joint without subluxation/dislocation.

Fourth, we report our case of 42-year-old gentleman with history of a heavy object falling over his left foot. On examination, we found bony prominence over the plantar aspect of foot in the region of the 3rd MTP joint. Radiograph revealed dorsal dislocation of 3rd Metatarsophalangeal joint (Fig. 1). Closed reduction was performed by giving axial traction and dorsally directed pressure over the plantar aspect of the head of the 3rd metatarsal. Buddy strapping of 3rd and 2nd toes was done. A slab was applied. After six weeks, the slab was removed and full weight bearing mobilisation permitted. Follow-up radiographs showed 3rd MTP joint congruity and alignment to be normal (Fig. 2a, 2b). At one year follow-up, the patient did not have any complaints of pain in the region and had no instability/recurrent dislocation. Isolated 3rd MTP joint dislocation is extremely rare. To the best of our knowledge, this is the first such reported case.
